# Intravenous Administration of Human Adipose Derived-Mesenchymal Stem Cells Is Not Efficient in Diabetic or Hypertensive Mice Subjected to Focal Cerebral Ischemia

**DOI:** 10.3389/fnins.2019.00718

**Published:** 2019-07-16

**Authors:** Gabrielle Mangin, Adrien Cogo, Anaïck Moisan, Philippe Bonnin, Benjamin Maïer, Nathalie Kubis, on behalf of the RESSTORE Consortium

**Affiliations:** ^1^INSERM, U965, CART, Paris, France; ^2^INSERM, U1148, Laboratory for Vascular and Translational Science, Universite de Paris, Paris, France; ^3^Unité de Thérapie et d’Ingénierie Cellulaire, EFS Auvergne Rhône Alpes, Saint-Ismier, France; ^4^Service de Physiologie Clinique-Explorations Fonctionnelles, AP-HP, Hôpital Lariboisière, Paris, France

**Keywords:** stroke, cell therapy, adipose tissue, cognitive decline, inflammation, behavior

## Abstract

As the second cause of death and cognitive decline in industrialized countries, stroke is a major burden for society. Vascular risks factors such as hypertension and diabetes are involved in most stroke patients, aggravate stroke severity, but are still poorly taken into account in preclinical studies. Microangiopathy and sustained inflammation are exacerbated, likely explaining the severity of stroke in those patients. We sought to demonstrate that intravenous administration of human adipose derived-mesenchymal stem cells (hADMSC) that have immunomodulatory properties, could accelerate sensorimotor recovery, prevent long-term spatial memory impairment and promote neurogenesis, in diabetic or hypertensive mice, subjected to permanent middle cerebral artery occlusion (pMCAo). Diabetic (streptozotocin IP) or hypertensive (L-NAME in drinking water) male C57Bl6 mice subjected to pMCAo, were treated by hADMSC (500,000 cells IV) 2 days after cerebral ischemia induction. Infarct volume, neurogenesis, microglial/macrophage density, T-lymphocytes density, astrocytes density, and vessel density were monitored 7 days after cells injection and at 6 weeks. Neurological sensorimotor deficit and spatial memory were assessed until 6 weeks post-stroke. Whatever the vascular risk factor, hADMSC showed no effect on functional sensorimotor recovery or cognitive decline prevention at short or long-term assessment, nor significantly modified neurogenesis, microglial/macrophage, T-lymphocytes, astrocytes, and vessel density. This work is part of a European program (H2020, RESSTORE). We discuss the discrepancy of our results with those obtained in rats and the optimal cell injection time frame, source and type of cells according to the species stroke model. A comprehensive understanding of the mechanisms preventing recovery should help for successful clinical translation, but first could allow identifying good and bad responders to cell therapy in stroke.

## Introduction

Stroke is currently the second cause of death and cognitive decline worldwide (Snyder et al., 2015; World Health Organization [WHO], 2018). Recanalization treatments, either by rtPA (recombinant tissue plasminogen activator) or thrombectomy, can only be used at the acute phase, with less than 10% of patients being concerned (Bhaskar et al., 2018). In that context, cell therapy, using stem cells/ progenitors or differentiated cells, was developed in order to potentially repair tissue damage and improve neurological outcome taking advantage of a wider therapeutic window (Mangin and Kubis, 2018).

Among them, mesenchymal stem cells (MSCs) are particularly attractive. They are multipotent cells capable of differentiating into at least one differentiated cell type depending on environmental conditions (Chamberlain et al., 2007). These cells improve neurological deficit after stroke in both mice (Chen et al., 2003) and rats (Li et al., 1999; Cho et al., 2012) but their mechanism of action is still poorly understood, although neurogenesis, angiogenesis and immunomodulation, all contributing to new neuronal networks formation, have been associated to improved neurological recovery (Chen et al., 2014).

Mesenchymal cells derive from multiple sources such as bone marrow, cord blood, or adipose tissue, this last one offering numerous advantages. A large number of cells can be easily collected by liposuccion. Expansion of cells is easier than when MSC are derived from bone marrow (BM-MSC), the other largest source of MSC and they could be more effective than BM-MSC (Mangin et al., 2019). Indeed, they proliferate better *in vitro* and further reduce the cerebral ischemic-related damage in a mouse model of transient middle cerebral artery occlusion (tMCAo) compared to cells isolated from bone marrow (Ikegame et al., 2011). In addition, they showed an excellent safety profile in 8 clinical trials representing 321 patients (Lalu et al., 2012) and their low immunogenicity after allogeneic administration (Pers and Jorgensen, 2013) make them a likely “ready-to-use” therapeutic product.

Up to date, few studies have considered the impact of vascular risk factors on stroke outcome. This is quite surprising since hypertension, the first leading vascular risk factor for stroke, is still dramatically underdiagnosed, with only 32.5% of hypertensive patients being pharmacologically treated and controlled (Chow et al., 2013). Diabetes is another independent risk factor for stroke, tripling its incidence (Mankovsky and Ziegler, 2004). These data are particularly alarming since the diabetic population will double by 2030 (Shaw et al., 2010). These two vascular risk factors are associated with increased disability, death (Knopman et al., 2001; Ettehad et al., 2016) and post-stroke cognitive decline (Gorelick et al., 2011). As hypertensive and diabetic patients are at higher risk of stroke and therefore potential candidates for cell therapy, understanding how vascular risk factors influence donors’ cells properties and efficiency is therefore mandatory (Mangin and Kubis, 2018).

This work if part of a H2020 program (RESSTORE) that includes clinical and preclinical work-packages, and whose aim is to assess the efficiency of cell therapy in stroke taken into account vascular risk factors. Preclinical evaluation complied with the STAIRS (Liu et al., 2009) and STEPS II (Savitz et al., 2011) recommendations. Mesenchymal stem cells derived from human adipose tissue (hADMSC) obtained from healthy donor, were injected intravenously 2 days after induction of a focal cerebral ischemia in mice in which hypertension or diabetes had been previously induced. The primary objective of our work was to evaluate the short (1 week) and long-term (6 weeks) consequences on neurological sensorimotor and cognitive decline and on stroke volume. Neurogenesis, inflammation, and angiogenesis were secondarily evaluated.

## Materials and Methods

All experiments and surgical procedures were performed according to the European Community Directive (2010/63/EU), the ARRIVE (Animal Research Reporting In Vivo Experiments) guidelines, and the French national guidelines for the care and use of laboratory animals. The study was specifically approved by our local institutional ethics committee (CE121#/17581v6) and by the French ministry of Higher Education for Research and Innovation (APAFIS#5431-2016031912549126 v2).

### Experimental Design

**FIGURE 1:**
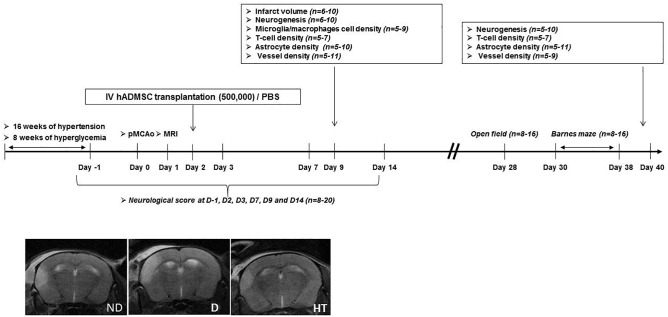
Experimental protocol and time schedule for hypertensive and diabetic mice. hADMSC, human adipose derived mesenchymal stem cells; pMCAo, permanent, middle cerebral artery occlusion; n, number of mice.

The experimental design is summarized in Figure 1. Experiments were performed on male adult C57/BL6 mice (Janvier Labs, Le Genest-Saint-Isle, France) housed in a 12 h/light/dark cycle with food and water *ad libitum*. Prior to stroke, either hypertension or diabetes was induced. The duration of sustained hyperglycemia and elevated blood pressure were determined to be the minimal duration to induce an increased sensorimotor deficit compared to age-matched mice without vascular risk factors. Because timing differed between diabetic (2 months) and hypertensive mice (4 months), control groups were matched with age. At day 0 (D0), permanent occlusion of middle cerebral artery was performed. At day 1 (D1), mice undergone cerebral MRI to be randomized in treated or untreated groups, according to the infarct volume and IV hADMSC or PBS were intravenously (IV) delivered the day after (D2). Sensorimotor evaluation was conducted from D-1 until D14, open field at D28 and cognitive assessment between D30 and D38. Stroke volume measured after Cresyl violet staining was assessed at day 9 (7 days after hADMSC delivery) and neuroblasts density, microglia/macrophages cells density, T-Lymphocytes density, astrocytes density, and vessels density assessment were performed 1-week post-hADMSC delivery (9 days post-stroke, D9) or 40 days post-stroke (D40), i.e., at the end of the cognitive assessment period, to evaluate the histological consequences of hADMSC injection. Thus, a subset of mice was sacrificed at D9 and another at D40 for histological and immunohistochemical analyses.

The following groups were evaluated: untreated non-diabetic (ND-PBS) and diabetic (D-PBS) mice and treated non-diabetic (ND-hADMSC) and diabetic (D-hADMSC) mice; untreated non-hypertensive (NHT-PBS) and hypertensive (HT-PBS) mice and treated non-hypertensive mice (NHT-hADMSC) and hypertensive mice (HT-hADMSC).

In a separate set of diabetic and hypertensive mice, vasoreactivity was assessed after 8 weeks of diabetes and hypertension induction, by measuring with Doppler ultrasound the variations of mean blood flow velocity at the basilar trunk after CO_2_ inhalation.

All experiments were conducted by investigators blinded to the status (diabetic and non-diabetic, hypertensive, and non-hypertensive) and the treatment (hADMSC or PBS).

### Diabetes Induction

At 6 weeks of age, C57Bl6/J mice were randomized to receive for five consecutive days intraperitoneal (IP) injections of STZ (60 mg/kg in 100 μL of citrate buffer) to induce diabetes or 100 μL of citrate buffer for non-diabetic mice. To ensure that mice became diabetic, blood glucose levels were monitored once a week by collecting blood through a skin incision at the tail. Only mice with sustained hyperglycemia above 300 mg/dL were considered as diabetic (90% of treated mice).

### Hypertension Induction

At 6 weeks of age, C57Bl6/J mice were randomized to receive during 16 weeks L-NAME [*N*(G)-nitro-L-arginine methyl ester, 60 mg/kg/day] in drinking water to induce hypertension, or without L-NAME for the non-hypertensive mice. Systolic and diastolic blood pressure and heart rate were measured after 2 weeks of habituation once a week until sacrifice, using a tail-cuff plethysmograph connected to a computerized system (BP-2000 computerized system, Visitech Systems, Apex, NC, United States). Measurements were performed in vigil mice, placed on a heating blanket. All mice became hypertensive.

### Doppler Imaging of Cerebral Vasoreactivity to Inhaled CO_2_

Cerebral vasoreactivity was assessed in all groups after 8 weeks of hyperglycemia and hypertension induction as previously described (Poittevin et al., 2015). This time point was previously determined in preliminary studies to be the shortest time to impair vasoreactivity. Ultrasound measurements were performed under 0.5% isoflurane anesthesia, using an echocardiograph (Acuson S3000, Siemens, Erlangen, Germany), equipped with a 14-MHz probe (14L5 SP). As previously described, time-averaged mean blood flow velocities were measured at the basilar trunk under air and after 5 min of breathing a gas mixture of 16% O_2_, 5% CO_2_, and 70% N_2_.

### Permanent Middle Cerebral Artery (pMCAo) Induction

This procedure has already been fully described (Poittevin et al., 2013). Briefly, at D0, mice were anesthetized by isoflurane (initially 2%, followed by 1.5 to 1.8% in O_2_) and body temperature was continuously maintained at 37 ± 0.5°C during the whole procedure using a heating blanket (Homeothermic Blanket Control Unit; Harvard Apparatus Limited, United Kingdom). The permanent focal cerebral ischemia was induced by electrocoagulation of the left middle cerebral artery. The whole procedure did not exceed 10 min.

### Cerebral MRI

MR examinations were performed at D1 at the FRIM plateform using a 7.0 Tesla MR unit (PharmaScan, Bruker Biospin, Ettlingen, Germany) equipped with a surface coil with an internal diameter of 10 mm, in thermoregulated and anesthetized (2% isoflurane in O_2_) mice. T2-weighted imaging [Rapid Imaging with Refocused Echoies (RARE) sequence was used with the following parameters: effective TE: 33 ms, TR: 2742 ms, NEX: 1, Flip angle: 180°] was performed in order to measure infarct volume (Medical Image Processing, Analysis and Visualization or MIPAV software, NIH, Bethesda, MD, United States) and T2^∗^-weighted imaging [a Fast Low Angle Shot (FLASH) sequence was used with the following parameters: 15 axial slices 1 mm thick, TE: 8 ms, TR: 600 ms, NEX: 2, Flip angle: 30°, FOV: 2 cm × 2 cm, MTX: 256 × 256] to verify that there was no hemorrhage in any of these mice.

### hADMSC Collection

Adipose tissue was harvested from the subcutaneous abdominal adipose tissue by lipo-aspiration from one young healthy female then transferred to the cell therapy unit for digestion. Negativity of the donor for HIV1/2, HBV, HCV, HTLV1/2, HSV1/2, Syphilis, CMV, and EBV serology was verified in order to be compliant with the Directive 2006/17/EC. At the end of the aspiration procedure, adipose tissue was transferred to the Etablissement Français du Sang (EFS) cell therapy unit for digestion according to an internal procedure. The adipose tissue stem cell line RESSTORE01 (Master Cell Bank/Stock no. 1—Donor RESSSTORE01, Batch no. 591133643763) was cultured in the growth medium Alpha MEM (Macopharma) supplemented with 5% human platelet lysate (Stemulate, Cook Medical, United States). The medium was changed twice each week and cells were passaged when they reached (85%) confluence. The cells were detached with TrypLE Select (Life Technologies) for 7–10 min. The RESSTORE01 cell phenotype was analyzed with flow cytometry (FACS Canto II, BD Biosciences) at passage P1. The BD Stemflow hMSC Analysis kit (ref: 562245, Becton Dickinson) was used to phenotype the ADSC that were characterized by the expression of the surface markers CD73, CD44, CD105, and CD90. In addition, in order to identify non-mesenchymal cell contamination, specific markers of monocytes/macrophages (CD11b), lymphocytes (CD19), pan-leucocytes (CD45), hematopoietic stem cells (CD34), and HLA-DR were performed. The expression of these positive markers should be ≥90% and ≤5% for the negative markers. These specifications have been established based on the recommendations of the International Society for Cellular Therapy. These hADMSC were harvested after expansion in MEMα medium (Thermofisher) and human platelet lysate 5% (Cook Regentec). At D2 post-stroke 500,000 cells in 0.2 mL PBS were administered through the retroorbital vein.

### Sensorimotor Deficit Assessment

Mice underwent a neurological evaluation consisting of five tests: neurological score, grip and string test, beam walking test and pole test, currently used in our laboratory (Poittevin et al., 2013, 2015; Hilal et al., 2018) and that have been fully described elsewhere (Frechou et al., 2019). The maximum global score was 19, with the lower neurologic score corresponding to a more severe deficit. The assessment was performed at D-1 (habituation stage, the mouse being its own control), D2 (just before cell infusion), D3, D7, D9, and D14 in all groups.

### Cognitive Assessment

Before testing spatial memory (Barnes maze), we evaluated the sensorimotor deficit as well as spontaneous locomotor activity (open field) at D28, since residual neurological sensorimotor deficit or anxiety could otherwise introduce a bias in the interpretation of the Barnes maze. Cognitive assessment was performed in a dedicated room where mice were placed 2 weeks before in order to acclimate to the room where they were subjected to a 12 h night-day cycle with food and water provided *ad libitum*.

#### Open Field

Residual sensorimotor deficit and potential anxiety likely to cause the mice to “freeze,” could interfere with the Barnes maze evaluation thus skewing the results. We used the open-field as previously described (Cifuentes et al., 2015). Spontaneous activity (distance traveled in cm and mean velocity in cm/s) was measured automatically for 30 min by an EthoVision XT system and analyzed by EthoVision XT 11.5 tracking software (Noldus, Wageningen, Netherlands). Open field activity was assessed at D28 in all groups that were then tested on the Barnes Maze platform.

#### Barnes Maze

The Barnes maze is an open space 1 m in diameter, consisting of 20 holes spaced equidistantly around the circumference; one escape box is placed under one of the holes that the mouse will learn to find using visual cues placed in the behavior room. Mice are trained three times a day with a 15-min rest between each trial and recorded with a camera.

As previously described (Cifuentes et al., 2017), at D30, the mouse has to learn the location of the escape box on four consecutive days, named the training phase (1^st^ day to 4^th^ day of evaluation). After 2 days of resting (7^th^ day of evaluation), mice are tested on their ability to recall the place of the escape box, named the retention phase. The next day (8^th^ day), the escape is box is changed from its original location and mice have to adapt, learn and memorize the new location during 2 days (8^th^ day and 9^th^ day). During this last phase or reversal phase, brain plasticity is evaluated. During each phase, the latency to escape (seconds) is measured.

### Assessment of Infarct Volume

At D9, 1 week after hADMSC IV administration, mice were transcardiacally perfused with heparinized saline, followed by 4% paraformaldehyde (PFA). Brain was removed, post-fixed overnight in PFA and cryoprotected in 20% sucrose. Coronal 30 μm-thick sections were cut frozen using a cryostat CM 1950 (Leica Biosystems, Nußloch, Germany). Every eight 30 μm-thick floating section was stained with Cresyl violet. Using ImageJ software (National Institutes of Health, Bethesda, MD, United States), the cortical infarct area was measured to calculate the stroke volume (mm^3^) by integrating measured areas and distances between sections. Measured infarct volume was corrected for edema with the following formula: corrected infarct volume = measured infarct volume × (contralateral hemisphere volume/ipsilateral hemisphere volume) as already described (Poittevin et al., 2013).

### Assessment of Histological Response to hADMSC

Thirty μm-thick coronal floating sections were incubated with primary antibody overnight at 4°C to detect neuroblasts using anti-doublecortin (DCX) (1:4000, Abcam, Cambridge, United Kingdom); microglial cells using anti-Iba1 (1:200, Wako, Japan), T-lymphocytes using anti-CD3 (1/200, Dako, Santa Clara, United States), astrocytes using anti-GFAP (1:200, Millipore, Burlington, MA, United States) and vessels using the anti-glucose transporter-1 (Glut-1) (1:500, Millipore, Burlington, MA, United States). Appropriate fluorescent-labeled secondary alexa fluor 594 or 488 antibodies (Molecular Probes, Eugene, OR, United States 1:400) were applied for 1 h at room temperature. Specificity was checked by omitting the primary antibody. As doublecortin is expressed in newborn and migrating neurons, we assumed that DCX+ cells reflect neurogenesis (Brown et al., 2003; Jin et al., 2010).

### Morphological Analysis

Cells density assessments were conducted blindly (vascular risk factor and treatment) at D9 and D40 and performed on three coronal brain sections at +0.80, −0.80, and −1.20 mm relative to bregma, that consistently contained the infarct area.

*Neuroblasts density* was assessed by calculating the DCX-positive area (X10) using NIH ImageJ software, in the whole ipsilateral hemisphere to stroke, actually only found in the subventricular zone and along the corpus callosum along which they migrate toward the lesion.

*Microglial/macrophage* density was assessed by calculating the Iba1-positive area (X5) using NIH ImageJ software in the peri-infarct area.

*T-cell* density was manually assessed by counting CD3+ cells in each section, actually only present in the infarct area, and expressed as the number of CD3+ cells per section.

*Astrocytes* density was assessed by calculating the GFAP-positive area in 2 R.O.I (X40) using NIH ImageJ software in the peri-infarct area.

*Vessel* density was assessed by calculating the GLUT-1-positive area (X20) using NIH ImageJ software in the peri-infarct area.

All calculated areas were expressed as arbitrary units.

### Statistical Analysis

Statistical analyses were performed using Prism 7.02 software (GraphPad, San Diego, CA, United States). The data are expressed in mean ± SD. The Shapiro–Wilk normality test was used to assess whether the data corresponded to a Gaussian distribution. Comparisons between the four groups of diabetic mice or of hypertensive mice were analyzed using either ANOVA or a Kruskal–Wallis test. Two-way ANOVA for repeated measures was used to analyze data of the training (1^st^ to 4^th^ days) and reversal (8^th^ to 9^th^ days) phases in the Barnes maze. *Post hoc* analyses were performed using Bonferroni’s multiple comparison tests. A value of *p* < 0.05 was considered statistically significant.

## Results

Mean glycemia was not significantly different at D-1 between D-PBS (451.7 ± 59.7 mg/dl) and D-hADMSC mice (431.8 ± 50.1 mg/dl). No mice died whatever the group during surgery and during follow-up. hADMSC did not significantly modify glycemia in diabetic mice during follow-up until D40 (460.3 ± 56.6 mg/dl) compared to untreated D-PBS mice (493.3 ± 70.9 mg/dl) (*n* = 13–14).

Before stroke, systolic and diastolic blood pressure were significantly higher in HT mice (134.9 ± 5.9/89.0 ± 7.2 mmHg) compared to NHT mice (114.0 ± 7.0/78.6 ± 6.2 mmHg) (*p* < 0.0001). After stroke and during follow-up, hADMSC did not significantly modify systolic blood pressure (NHT-PBS: 104.9 ± 14.1 mmHg vs. NHT-hADMSC 111.7 ± 6.3 mmHg; HT-PBS: 138.3 ± 7.2 mmHg vs. HT-hADMSC: 136.2 ± 11.2 mmHg), or diastolic blood pressure (NHT-PBS: 82.1 ± 9.5 mmHg vs. NHT-hADMSC: 80.9 ± 6.5 mmHg; HT-PBS: 86.3 ± 7.3 mmHg vs. HT-hADMSC: 90.1 ± 6.6 mmHg). Heart rate (beats per min) did not differ significantly between groups (NHT-PBS: 536 ± 10 vs. NHT-hADMSC: 522 ± 17 vs. HT-PBS: 518 ± 36 vs. HT-hADMSC 507 ± 35) (*n* = 18–20). No mice died whatever the group during surgery and during follow-up.

### Doppler Imaging of Cerebral Vasoreactivity to Inhaled CO_2_

Cerebral vasoreactivity was assessed 8 weeks after the induction of chronic hyperglycemia or chronic hypertension, before cerebral ischemic induction. Mean blood flow velocities at the basilar trunk were not significantly modified after CO_2_ inhalation in diabetic mice (air: 12.0 ± 1.7 versus CO_2_: 11.0 ± 1.4 cm/s) (*n* = 7) and in hypertensive mice (air: 15.7 ± 2.2 versus CO_2_: 15.8 ± 2.6 cm/s) (*n* = 14), whereas they were significantly increased when compared to the same control mice (non-diabetic and non-hypertensive mice) (air: 15.4 ± 2.4 versus CO_2_: 17.7 ± 2.2 cm/s, *p* = 0.0001) (*n* = 9), indicating that in this last group only, CO_2_ induced arteriolar vasodilation.

### Sensorimotor Deficit

**FIGURE 2:**
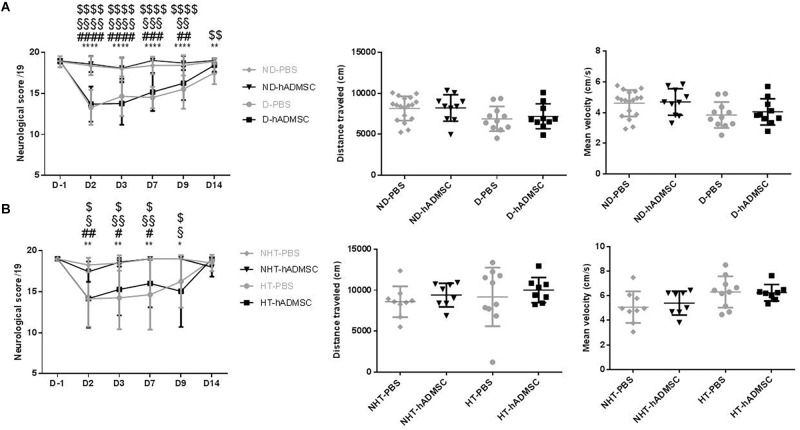
Assessment of sensorimotor deficit between D-1 and D14 and of spontaneous locomotor activity measured at D28 in ND-PBS, untreated non-diabetic mice; ND-hADMSC, treated non-diabetic mice; D-PBS, untreated diabetic mice and D-hADMSC, treated diabetic mice (*n* = 13–20) **(A)** and NHT-PBS, untreated non-hypertensive mice; NHT-hADMSC, treated non-hypertensive mice; HT-PBS, untreated hypertensive mice and HT-hADMSC, treated hypertensive mice (*n* = 8–20) **(B)**. Diabetic and hypertensive mice showed a more severe sensorimotor deficit than control mice during the whole evaluation until they fully recovered at D14 except D-PBS mice (*p* < 0.01 versus ND-PBS and *p* < 0.01 versus ND-hADMSC). No differences were evidenced between untreated and treated mice. At D28, no differences were evidenced between mice in the total distance traveled or mean velocity at open field; ^∗^*p* < 0.05, ^∗∗^*p* < 0.01, ^∗∗∗∗^*p* < 0.0001 between ND-PBS versus D-PBS mice and NHT-PBS versus HT-PBS mice; ^#^*p* < 0.05, ^##^*p* < 0.01, ^###^*p* < 0.001, ^####^*p* < 0.0001 between ND-PBS versus D-hADMSC and between NHT-PBS versus HT-hADMSC; ^§^*p* < 0.05, ^§§^*p* < 0.01, ^§§§^*p* < 0.001, ^§§§§^*p* < 0.0001 between ND-hADMSC versus D-PBS and between NHT-hADMSC versus HT-PBS; ^$^*p* < 0.05, ^$$^*p* < 0.01, ^$$$$^*p* < 0.0001 between ND-hADMSC versus D-hADMSC and NHT-hADMSC versus HT-hADMSC.

At D2, compared to D-1, we observed a significant reduction in the neurological score in diabetic and hypertensive mice compared to control non-diabetic and non-hypertensive mice, indicating that both diabetes and hypertension induced a more severe sensorimotor deficit compared to mice that were subjected to the same pMCAo stroke model but without vascular risk factor. From D2 to D9, this deficit significantly persisted over time until complete recovery by D14, except in D-PBS that had nevertheless completely recovered by D28. However, whatever the time of evaluation, there was no significant difference in the neurological score between untreated and treated mice whatever the risk factor, indicating that hADMSC had no effect on sensorimotor recovery (Figure 2A,B and Tables 1, 2).

**Table 1:** Sensorimotor assessment in diabetic mice.

Day	ND-PBS (*n* = 20)	ND-hADMSC (*n* = 16)	D-PBS (*n* = 14)	D-hADMSC (*n* = 13)
D-1	18.85 ± 0.67	18.94 ± 0.25	19.00 ± 0.00	18.92 ± 0.28
D2	18.35 ± 1.14	18.56 ± 0.96	13.29 ± 2.13	13.69 ± 2.14
D3	18.00 ± 1.41	18.06 ± 1.29	14.64 ± 2.41	13.77 ± 2.59
D7	18.40 ± 1.08	19.00 ± 0.00	14.50 ± 1.31	15.17 ± 2.38
D9	18.40 ± 1.23	18.69 ± 0.87	15.50 ± 2.38	16.23 ± 2.048
D14	18.90 ± 0.32	19.00 ± 0.00	17.50 ± 1.38	18.43 ± 0.79

*Control diabetic (D-PBS) and non-diabetic (ND-PBS) mice, treated diabetic (D-hADMSC), and non-diabetic mice (ND-hADMSC).*

**Table 2:** Sensorimotor assessment in hypertensive mice.

Days	NHT-PBS (*n* = 8)	NHT-hADMSC (*n* = 10)	HT-PBS (*n* = 20)	HT-hADMSC (*n* = 18)
D-1	19.00 ± 0,00	18.89 ± 0.33	19.00 ± 0.00	19.00 ± 0.00
D2	18.25 ± 0.89	17.44 ± 1.24	14.15 ± 3.41	14.17 ± 3.55
D3	18.50 ± 0.93	18.60 ± 0.52	14.25 ± 3,82	15.28 ± 3.16
D7	19.00 ± 0.00	19.00 ± 0.00	14.65 ± 4.27	16.00 ± 2.89
D9	19.00 ± 0.00	19.00 ± 0.00	16.25 ± 3.19	15.50 ± 4.32

*Control hypertensive (HT-PBS) and non-hypertensive mice (NHT-PBS) and treated hypertensive (HT-hADMSC) and non-hypertensive mice (NHT-hADMSC).*

### Open Field

The ability of mice to spontaneously walk the equivalent distance with similar velocity was verified in the open field, before the Barnes Maze evaluation. Indeed, any residual motor impairment could skew the interpretation of spatial long-term memory. No differences were observed between mice in the distance traveled or mean velocity in the diabetic groups or in the hypertensive groups, whether the mice were treated or untreated (Figure 2A,B).

### Barnes Maze

**FIGURE 3:**
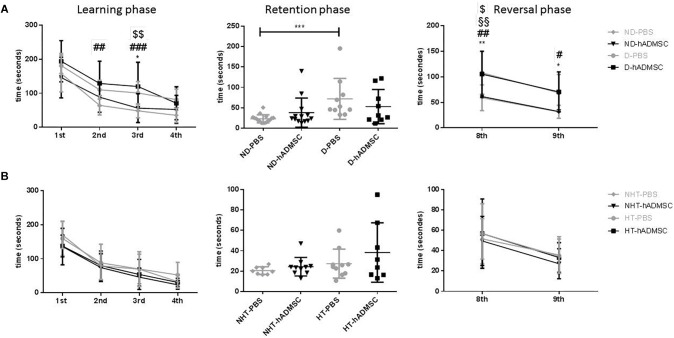
Assessment of long-term spatial memory between D30 and D38 in ND-PBS, untreated non-diabetic mice; ND-hADMSC, treated non-diabetic mice; D-PBS, untreated diabetic mice and D-hADMSC, treated diabetic mice (*n* = 9–16) **(A)** and NHT-PBS, untreated non-hypertensive mice; NHT-hADMSC, treated non-hypertensive mice; HT-PBS, untreated hypertensive mice and HT-hADMSC, treated hypertensive mice (*n* = 8–10) **(B)**. hADMSC did not significantly modify the latency to escape of pMCAo mice whatever the risk factor; ^∗^*p* < 0.05, ^∗∗^*p* < 0.01, ^∗∗∗^*p* < 0.001, ND-PBS versus D-PBS; ^#^*p* < 0.05, ^##^*p* < 0.01, ^###^*p* < 0.001, ^####^*p* < 0.0001 ND-PBS versus D-hADMSC; ^§^*p* < 0.05, ^§§^*p* < 0.01, ^§§§^*p* < 0.001, ^§§§§^*p* < 0.0001 ND-hADMSC versus D-PBS; ^$^*p* < 0.05, ^$$^*p* < 0.01, ^$$$^*p* < 0.001, ^$$$$^*p* < 0.0001 ND-hADMSC versus D-hADMSC.

In the training phase, D-hADMSC had an impaired learning ability at the 2^nd^ and 3^rd^ day compared to ND-PBS (*p* < 0.01 and *p* < 0.001, respectively). At the 3^rd^ day, D-PBS also presented an impairment compared to ND-PBS (*p* < 0.05) and D-hADMSC compared to ND-hADMSC (*p* < 0.01). However, all mice learned the same on the 4^th^ day. In the retention phase, only diabetic mice without treatment showed a delayed latency to escape reflecting an impaired long-term memory ability (ND-PBS versus D-PBS, *p* < 0.001). D-hADMSC did not show differences compared to non-diabetic mice, nor with D-PBS mice. On the reversal phase, all diabetic mice had an impaired cerebral plasticity compared to non-diabetic mice (ND-PBS versus D-PBS, *p* < 0.01; ND-PBS versus D-hADMSC, *p* < 0.01; ND-hADMSC versus D-PBS, *p* < 0.01; ND-hADMSC versus D-hADMSC, *p* < 0.05). This impairment was also present on the 9^th^ day for D-PBS mice and D-hADMSC compared to ND-PBS mice (*p* < 0.05) (Figure 3A and Table 3).

**Table 3:** Barnes maze in diabetic mice.

Days	ND-PBS (*n* = 16)	ND-hADMSC (*n* = 13)	D-PBS (*n* = 10)	D-hADMSC (*n* = 9)
1^st^	157.80 ± 53.71	146.00 ± 59.73	181.20 ± 35.50	194.00 ± 60.40
2^nd^	64.60 ± 28.10	88.63 ± 44.34	110.50 ± 27.29	128.90 ± 65.16
3^rd^	48.35 ± 20.47	56.46 ± 42.37	101.00 ± 33.31	119.90 ± 70.87
4^th^	35.26 ± 20.97	52.70 ± 40.81	81.38 ± 30.90	70.76 ± 48.27
7^th^	23.23 ± 9.85	38.29 ± 35.77	71.80 ± 50.08	53.02 ± 41.96
8^th^	59.09 ± 25.23	61.35 ± 30.93	107.90 ± 41.58	105.30 ± 44.89
9^th^	31.53 ± 12.80	32.13 ± 17.95	72.71 ± 32.68	57.20 ± 43.07

*Control diabetic (D-PBS) and non-diabetic (ND-PBS) mice, treated diabetic (D-hADMSC), and non-diabetic mice (ND-hADMSC).*

To summarize, hADMSC injected intravenously 2 days after a focal cerebral ischemia failed to protect from post-stroke cognitive impairment in diabetic mice.

In hypertensive mice, we evidenced no differences in the training phase, the retention phase or the reversal phase between the four groups, indicating that 16 weeks of hypertension did not induce a worse post-stroke cognitive impairment compared to non-hypertensive mice subjected to a focal cerebral ischemia. As we have previously shown (Mangin et al., 2019) that pMCAo mice, without vascular risk factor, does not have significantly modified scores at Barnes Maze, our results would indicate that our model did not produce cognitive impairment in hypertensive mice. The effect of hADSMC could therefore not be evaluated (Figure 3B and Table 4).

**Table 4:** Barnes maze in hypertensive mice.

Days	NHT-PBS (*n* = 9)	NHT-hADMSC (*n* = 10)	HT-PBS (*n* = 10)	HT-hADMSC (*n* = 8)
1^st^	170.20 ± 40.06	135.80 ± 53.44	159.60 ± 50.13	137.40 ± 31.63
2^nd^	79.38 ± 40.17	74.10 ± 40.59	88.22 ± 54.67	80.18 ± 42.32
3^rd^	69.61 ± 51.14	46.22 ± 26.37	70.14 ± 43.29	54.19 ± 44.01
4^th^	33.09 ± 19.45	23.67 ± 12.43	52.63 ± 37.05	30.92 ± 12.06
7^th^	20.60 ± 3.76	24.46 ± 9.08	27.13 ± 14.28	38.32 ± 29.13
8^th^	51.53 ± 19.76	49.45 ± 24.09	56.59 ± 29.37	56.67 ± 34.05
9^th^	35.75 ± 17.88	27.12 ± 14.78	33.20 ± 14.60	34.47 ± 16.90

*Control hypertensive (HT-PBS) and non-hypertensive mice (NHT-PBS) and treated hypertensive (HT-hADMSC), and non-hypertensive mice (NHT-hADMSC).*

### Infarct Volume

**FIGURE 4:**
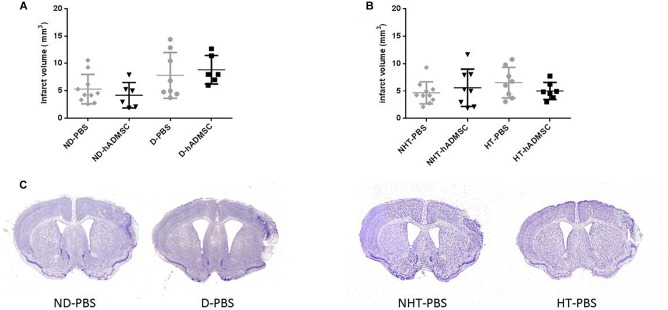
Assessment of infarct volume at D9 in ND-PBS, untreated non-diabetic mice; ND-hADMSC, treated non-diabetic mice; D-PBS, untreated diabetic mice and D-hADMSC, treated diabetic mice (*n* = 6–10) **(A)** and NHT-PBS, untreated non-hypertensive mice; NHT-hADMSC, treated non-hypertensive mice; HT-PBS, untreated hypertensive mice and HT-hADMSC, treated hypertensive mice (*n* = 7–10) **(B)**. hADMSC did not significantly reduce infarct volume whatever the risk factor. Representative crésyl violet sections of infarct areas for non-diabetic, diabetic, non-hypertensive, and hypertensive mice **(C)**.

Infarct volume was not significantly modified at day 9 in the diabetic groups or in the hypertensive groups, whether the mice were treated or untreated (Figure 4).

### Neurogenesis, Microglial/Macrophages, T Lymphocytes, Astrocytes, and Vessel Density and Were Not Significantly Modified by hADMSC in Diabetic and Hypertensive Mice

#### Neurogenesis

Comparison of the DCX-positive area was performed in order to assess neurogenesis between groups. At D9, no differences were evidenced between untreated and treated non-diabetic and diabetic mice and between untreated and treated non-hypertensive and hypertensive mice.

At D40, there was an overall ANOVA significance between untreated and treated non-diabetic and diabetic mice (*p* < 0.01). The DCX-positive area was significantly increased in D-hADMSC mice (1.6 ± 0.28 mm^3^) compared to ND-PBS mice (0.42 ± 0.40 mm^3^) (*p* < 0.001), whereas there was no significant difference with the other groups (1.01 ± 0.51 mm^3^, ND-hADMSC mice; 1.14 ± 0.57 mm^3^, D-PBS mice). No differences were evidenced between untreated and treated non-hypertensive and hypertensive mice.

**FIGURE 5:**
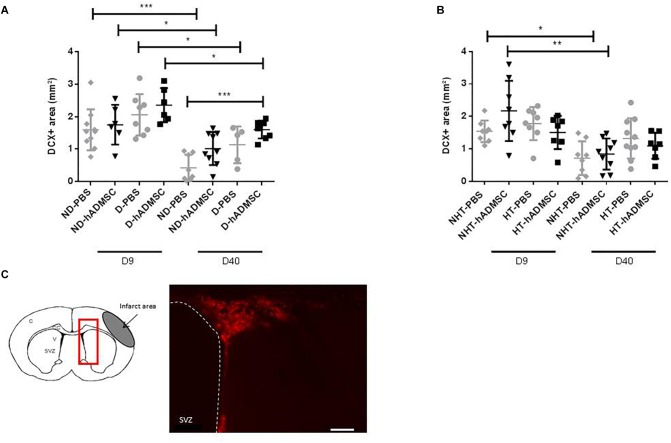
Assessment of neuroblasts density at D9 and D40 in ND-PBS, untreated non-diabetic mice; ND-hADMSC, treated non-diabetic mice; D-PBS, untreated diabetic mice and D-hADMSC, treated diabetic mice (D9: *n* = 6–10, D40: *n* = 5–9) **(A)** and NHT-PBS, untreated non-hypertensive mice; NHT-hADMSC, treated non-hypertensive mice; HT-PBS, untreated hypertensive mice and HT-hADMSC, treated hypertensive mice (D9: *n* = 7–8, D40: *n* = 7–10) **(B)**. hADMSC did not significantly increase neuroblasts density except at D40 in D-hADMSC mice compared to ND-PBS mice. Between D9 and D40, a significant decrease in neuroblasts density was evidenced in ND-PBS, *p* < 0.001; ND-hADMSC, *p* < 0.05; D-PBS, *p* < 0.05; D-hADMSC, *p* < 0.05) and in NHT-PBS (*p* < 0.05) and NHT-hADMSC (*p* < 0.01). ^∗^*p* < 0.05, ^∗∗^*p* < 0.01, ^∗∗∗^*p* < 0.001. DCX immunostaining in the SVZ of untreated diabetic mice, at D9 and at D40, scale bar: 50 μm; C, cortex; V, ventricle; SVZ, subventricular zone; red square, region of interest **(C)**.

Between D9 and D40, there was a significant reduction in neuroblasts density in ND-PBS (*p* < 0.001), ND-hADMSC (*p* < 0.05), D-PBS (*p* < 0.05) and D-hADMSC mice (*p* < 0.05), and in NHT-PBS (*p* < 0.05) and NHT-hADMSC mice (*p* < 0.01); no differences were observed in HT-PBS and HT-hADMSC mice over time (Figure 5).

#### Microglial/Macrophages Density

**FIGURE 6:**
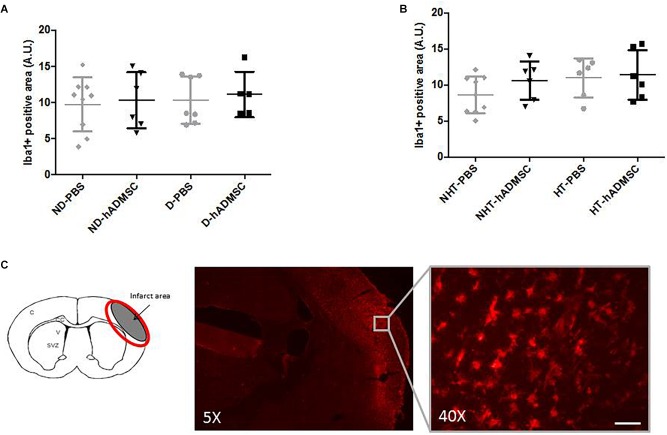
Assessment of Iba1+ cell density at D9 in ND-PBS, untreated non-diabetic mice; ND-hADMSC, treated non-diabetic mice; D-PBS, untreated diabetic mice and D-hADMSC, treated diabetic mice (*n* = 5–9) **(A)** and NHT-PBS, untreated non-hypertensive mice; NHT-hADMSC, treated non-hypertensive mice; HT-PBS, untreated hypertensive mice and HT-hADMSC, treated hypertensive mice (*n* = 6–9) **(B)**. No significant reduction in microglia/macrophages density by hADMSC was evidenced whatever the risk factor. Iba1 immunostaining in D-PBS mice with magnification showing amoeboid cells, scale bar: 50 μm; C, cortex; V, ventricle; SVZ, subventricular zone; red circle: region of interest **(C)**.

Comparison of the Iba1-positive area was performed in order to assess microglial/macrophages density between groups. Microglia/macrophage cells (Iba1+) adopted a round amoeboid shape in the peri-infarct area, indicating a change in the state of activation. At D9, no differences were evidenced between untreated and treated non-diabetic and diabetic mice or between untreated and treated non-hypertensive and hypertensive mice (Figure 6).

At D40, rare microglial/macrophages were evidenced on sections, and therefore no quantification was performed.

#### T Lymphocytes Density

Comparison of CD3+ cells density was performed in order to assess T lymphocytes density between groups. At D9, no differences were evidenced between untreated and treated non-diabetic and diabetic mice or between untreated or treated non-hypertensive or hypertensive mice.

At D40, no differences were evidenced between untreated and treated non-diabetic and diabetic mice or between untreated or treated non-hypertensive or hypertensive mice.

**FIGURE 7:**
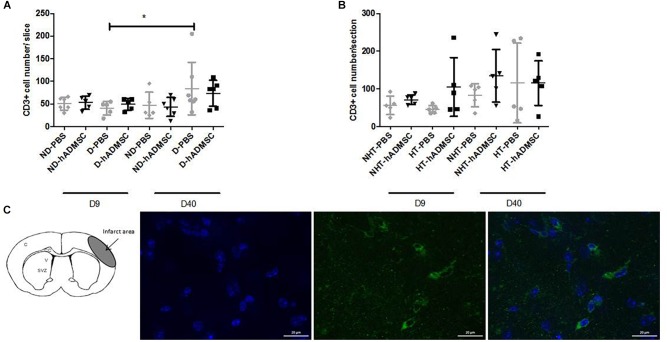
Assessment of CD3+ cells density at D9 and D40 in ND-PBS, untreated non-diabetic mice; ND-hADMSC, treated non-diabetic mice; D-PBS, untreated diabetic mice and D-hADMSC, treated diabetic mice (D9: *n* = 5–6, D40: *n* = 5–7) **(A)** and NHT-PBS, untreated non-hypertensive mice; NHT-hADMSC, treated non-hypertensive mice; HT-PBS, untreated hypertensive mice and HT-hADMSC, treated hypertensive mice (D9: *n* = 5–7, D40: *n* = 5) **(B)**. hADMSC did not significantly modify the T-lymphocytes density whatever the risk factor. Between D9 and D40, there was a significant increase in T-lymphocytes density in D-PBS mice (^∗^*p* < 0.05). From left to right, DAPI, CD3 and merge immunostaining, scale bar: 20 μm; C, cortex; V, ventricle; SVZ, subventricular zone **(C).**

Between D40 and D9, there was a significant increase in CD3+ cells density in D-PBS mice (84.3 ± 58/section versus 40.6 ± 15.0/section) (*p* < 0.05) only, whereas there were no significant differences in the CD3+ cells density between treated diabetic mice and untreated or treated non-diabetic mice. No differences were observed between untreated or treated non-hypertensive or hypertensive mice overtime (Figure 7).

#### Astrocytes Density

Comparison of the GFAP-positive area was performed in order to assess astrocytes density between groups. Activated astrocytes only were considered, when they adopted a narrow cell body and thickened their branches. At D9, no differences were evidenced between untreated and treated non-diabetic and diabetic mice. There was an overall significant difference between untreated or treated non-hypertensive or hypertensive mice (*p* = 0.02) and the GFAP-positive area was significantly decreased in HT-PBS (2.59 ± 0.35 A.U.) compared to NHT-PBS mice (8.06 ± 4.58 A.U.) (*p* < 0.05). No differences were evidenced with the other groups.

At D40, no differences were evidenced between untreated and treated non-diabetic and diabetic mice. There was an overall significant difference between untreated or treated non-hypertensive or hypertensive mice (*p* = 0.015) and the GFAP-positive area was significantly increased in HT-hADMSC mice (12.07 ± 2.43 A.U.) versus NHT-PBS mice (5.22 ± 4.52 A.U.) (*p* < 0.05), whereas no differences were evidenced with the other groups.

**FIGURE 8:**
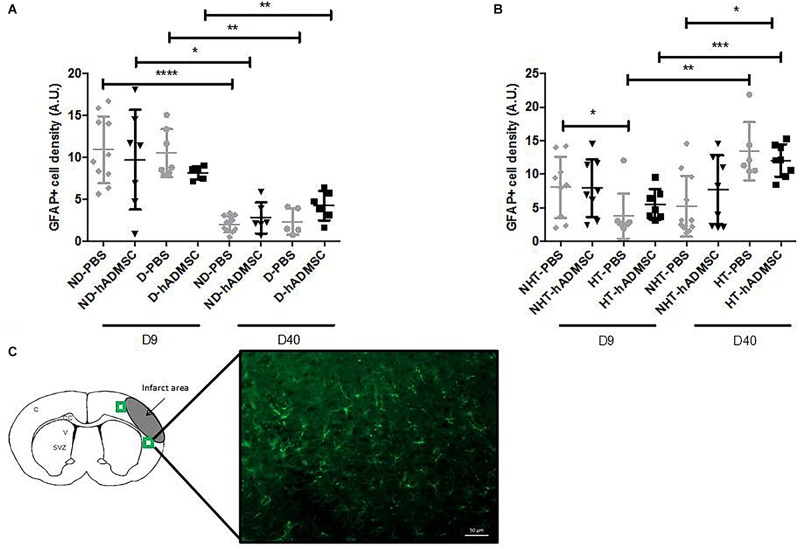
Assessment of GFAP+ cells density at D9 and D40 in ND-PBS, untreated non-diabetic mice; ND-hADMSC, treated non-diabetic mice; D-PBS, untreated diabetic mice and D-hADMSC, treated diabetic mice (D9: *n* = 5–10, D40: *n* = 5–11) **(A)** and NHT-PBS, untreated non-hypertensive mice; NHT-hADMSC, treated non-hypertensive mice; HT-PBS, untreated hypertensive mice and HT-hADMSC, treated hypertensive mice (D9: *n* = 7–9, D40: *n* = 6–11) **(B)**. hADMSC did not significantly modify the astrocytes density whatever the risk factor. Between D9 and D40, there was a significant reduction in activated astrocytes in ND-PBS, ND-hADMSC, D-PBS, and D-hADMSC mice and a significant increase in activated astrocytes in HT-PBS and HT-hADMSC mice; ^∗^*p* < 0.05, ^∗∗^*p* < 0.01, ^∗∗∗^*p* < 0.001, ^∗∗∗∗^*p* < 0.0001. GFAP immunostaining, scale bar: 50 μm; C, cortex; V, ventricle; SVZ, subventricular zone; green squares: regions of interest **(C).**

Between D40 and D9, the GFAP-positive area was significantly reduced in ND-PBS (2.04 ± 0.94 A.U. versus 10.90 ± 4.01 A.U.) (*p* < 0.0001), ND-hADMSC (2.79 ± 1.85 A.U. versus 9.72 ± 5.95 A.U.) (*p* < 0.05), D-PBS (2.34 ± 1.56 A.U. versus 10.49 ± 2.87 A.U.) (*p* < 0.01), and D-hADMSC (4.26 ± 1.74 A.U. versus 8.15 ± 0.80 A.U.) (*p* < 0.01) mice. By contrast, the GFAP-positive area was significantly increased in HT-PBS mice (13.47 ± 4.34 A.U. versus 2.59 ± 0.35 A.U.) (*p* < 0.01) and HT-hADMSC mice (12.07 ± 2.43 A.U. versus 5.49 ± 2.31 A.U.) (*p* < 0.001) (Figure 8).

#### Vessel Density

Comparison of the GLUT-1-positive area was performed in order to assess vessel density between groups. At D9 or at D40, no differences were evidenced between untreated and treated non-diabetic and diabetic mice or between untreated or treated non-hypertensive or hypertensive mice.

**FIGURE 9:**
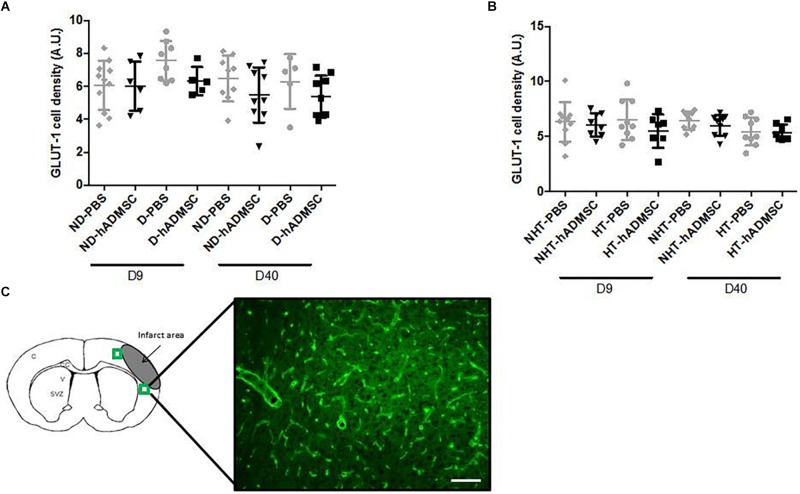
Assessment of GLUT-1+ cells area at D9 and D40 in ND-PBS, untreated non-diabetic mice; ND-hADMSC, treated non-diabetic mice; D-PBS, untreated diabetic mice; and D-hADMSC, treated diabetic mice (D9: *n* = 5–11, D40: *n* = 5–9) **(A)** and NHT-PBS, untreated non-hypertensive mice; NHT-hADMSC, treated non-hypertensive mice; HT-PBS, untreated hypertensive mice and HT-hADMSC, treated hypertensive mice (D9: *n* = 7–10, D40: *n* = 8–9) **(B)**. Vessel density was not significantly modified over time and hADMSC did not significantly modify the vessels density whatever the risk factor. GLUT-1 immunostaining, scale bar: 100 μm; C, cortex; V, ventricle; SVZ, subventricular zone; green squares: regions of interest **(C)**.

Between D9 and D40, no differences in the GLUT-1-positive area were evidenced between untreated and treated non-diabetic and diabetic mice or between untreated or treated non-hypertensive or hypertensive mice (Figure 9).

## Discussion

We showed that IV administration of hADMSC 2 days after focal cerebral ischemia induction did not significantly modify infarct volume, short and long-term functional outcome in diabetic or hypertensive mice neither was associated with significant modification of neurogenesis, inflammatory cells density or angiogenesis, whatever the analysis time frame.

Most of preclinical studies investigating the beneficial effect of cell therapy have been performed in healthy young male rodents, even when vascular risk factors are known to worsen the infarct lesion, functional outcome and mortality, and therefore question the efficiency of cell therapy. We have used a diabetic stroke model that we previously characterized (Poittevin et al., 2015). Streptozotocin induced chronic hyperglycemia was associated after 8 weeks to impaired vasoreactivity and decreased brain eNOS and nNOS expression. After stroke induction, diabetic mice had an increased sensori-motor disability (Poittevin et al., 2015) and presented cognitive impairment at D30 compared to non-diabetic mice (Mangin et al., 2019). For the hypertensive model, we used L-NAME in daily drinking water that induced chronic mild hypertension, rather than chronic infusion of angiotensin 2 that is responsible for a more severe hypertension and requires additional surgery for angiotensin 2 pump implantation (Cifuentes et al., 2015, 2017). This model was also associated with impaired vasoreactivity after 8 weeks, but to achieve a sensori-motor impairment in our experimental conditions, we had to administer L-NAME during 16 weeks.

Cell therapy in post-stroke diabetic rodents has already been investigated using various cell types, sources and administration pathways. Allogenic bone-marrow derived MSC (BM-MSC) from healthy rats have been intravenously injected 1 day after stroke in type 1 diabetic rats, and 3 days after stroke in type 2 diabetic rats subjected to tMCAo. In the first case, blood-brain-barrier permeability and mortality were increased with an opposite outcome in the second case (Chen et al., 2011; Ding et al., 2016). In type 2 diabetic rats, xenogenic human BM-MSC that were IV delivered 3 days after a tMCAo, significantly improved neurological function without affecting infarct volume. Endothelial progenitor cells (EPCs) isolated from human umbilical cord blood and IV delivered immediately after reperfusion in type 1 diabetic mice subjected to a tMCAo, reduced blood-brain barrier leakage, reduced infarct volume and attenuated neurological impairment (Geng et al., 2017). By contrast, intraarterial delivery of allogenic BM-EPC in type 2 diabetic mice, 24 h after induction of a photothrombotic stroke, failed to significantly improve behavioral scores and infarct volume (Bai et al., 2015). Diabetes models, stroke models, species, delay of injection after stroke and allograft versus xenograft differed between studies and with ours, precluding direct comparison. Interestingly, xenograft or delayed injection of BM-MSC, up to 3 days, in diabetic animals, was not an impediment to improved outcome. Cell therapy in hypertensive rodents have only been investigated in spontaneously hypertensive rats. BM-derived mononuclear cells did not succeed in demonstrating sensori-motor improvement when IV transplanted 1 h after induction of a photothrombotic stroke (Diederich et al., 2014) or IV transplanted 3 h after reperfusion in a tMCAo stroke model (Minnerup et al., 2014), nor did umbilical cord blood mononuclear cells (Weise et al., 2014). Only maternal placenta derived MSC that were intravenously administered, led to an improvement of functional outcome (Kranz et al., 2010). These mitigate results underline the necessity to integrate co-morbidity factors into stroke models.

The source of MSC may matter. Adipose tissue is an interesting alternative donor tissue to bone marrow. Lipoaspiration allows easy collection of great quantities of cells (Mangin and Kubis, 2018). In addition, and in a translational point of view, ADMSC have a low immunogenicity profile enabling allogeneic administration with good tolerance. When transplanted in a tMCAo model, allogenic ADMSC IV delivered soon after reperfusion, showed a remarkable attenuation of ischemic damage compared to BM-MSC treated mice (Ikegame et al., 2011). In rats, a beneficial effect of ADMSC was observed whatever the IV (Gutierrez-Fernandez et al., 2015) or IA (Huang et al., 2017) administration, in a pMCAo and tMCAo model, respectively. Interestingly, xenogeneic ADMSC or allogeneic ADMSC showed equal efficacy in terms of functional recovery and decreased ischemic brain damage when IV delivered 30 min after pMCAo (Gutierrez-Fernandez et al., 2015). To summarize, ADMSC seem to be more promising than BM-MSC, the xenograft and allograft are of equal efficacy, they have been constantly reported to improve functional outcome in rats and mice, the tMCAo stroke model being the most widely used. However, none of these animals had associated vascular risk factors and cells were constantly administered, within 24 h after cerebral ischemia induction, both conditions that might explain our negative results.

Our model is not questioned since, using the same pMCAo model in diabetic mice, we previously showed a dramatic functional improvement and a reduced infarct volume following treatment with the immunomodulatory drug glatiramer acetate (Mangin et al., 2019), with peripheral blood mononuclear cells pretreated with ephrin-B2 in diabetic (personal unpublished data) but also in non-diabetic mice (Hilal et al., 2018). In literature, cell therapy is mainly reported in tMCAo stroke models. However, the pMCAo model offers many advantages. The tMCAO stroke model in diabetic mice is associated with a high mortality after 7 days which prevents any further follow-up (Poittevin et al., 2015). We had previously shown that diabetic mice developed post-stroke dementia after pMCAo (Mangin et al., 2019), which was one of the functional outcomes we wanted to explore. The smaller infarct, restricted to the cortex was crucial in our model, because we did want to spare the hippocampus from the neuronal damage. Moreover, it was recently reported that even a minor stroke increases the incidence of post-stroke dementia and that diabetes is the only vascular risk factor identified (Pendlebury et al., 2019). Although the infarct is smaller in this pMCAo stroke model compared to the tMCAo model in mice, brain inflammation is exacerbated (Zhou et al., 2013) and even more in diabetic mice (Mangin et al., 2019) and in hypertensive rats (Moller et al., 2015). This is important to consider since the efficiency of hADMSC is related to their “immunomodulatory properties” (Leu et al., 2010; Melief et al., 2013). At last, “healthy” non-diabetic and non-hypertensive mice subjected to a pMCAo presented little sensorimotor deficit that recovered completely very quickly and prevented us to evaluate any effect of cells in those mice, but this was not the primary endpoint of our study.

We cannot rule out that a different culture procedure might have led to different results. The fact that hADMSC were collected from only one donor might question the efficiency of these cells. However, using the cells of the same donor, Mu et al. (2019) succeeded in demonstrating functional improvement in treated non-diabetic adult male Sprague Dawley rats, subjected to focal cerebral ischemia, and using the sticky label test. However, this does not exclude that there might be variability in the response that should be tested by comparing the efficiency of these cells between various donors. Also, the delay of injection might have been too late. In our previous cell therapy studies in pMCAO mice, we constantly IV transplanted the cells within the first 24 h after stroke induction (Nih et al., 2012; Hilal et al., 2018) and a beneficial effect was observed when ADMSC were IV (Gutierrez-Fernandez et al., 2015) or IA administered (Huang et al., 2017), 30 min and 24 h respectively after the cerebral ischemia induction. The 48 h delay was chosen in that particular study to get along with the parallel clinical trial that aimed to widen the therapeutic window and because mice were randomized according to the infarct volume determined at cerebral MRI the day before.

The discrepancies of our results between mice and those obtained in rats by the other teams of the RESSTORE project (Mu et al., 2019), could be related to species differences in the immune system. In an *in vitro* model of oxygen-glucose deprivation on primary cultures of neurons, astrocytes, microglia in rats, mice or humans, the cytokines levels were differently expressed between all three species (Du et al., 2017), or even may be present in one species and not in the other (Mestas and Hughes, 2004). MSC have been shown to secrete cytokines and growth factors that inhibit T cells proliferation, decrease TNFα secreted by dendritic cells (Uccelli et al., 2008) and at the same time polarize lymphocytes and microglial cells to an “anti-inflammatory” and beneficial phenotype (Ankrum et al., 2014). Although hADMSC have been reported to have a higher immunomodulatory capacity than BM-derived counterparts, including increase of IL-6 and TGF-β1 (Melief et al., 2013) and decrease of IL-18 (Leu et al., 2010), we did not evidence any modification of microglial/macrophages, T lymphocytes, or astrocytes densities between treated and untreated mice, when adding co-morbidity, but we did not measure brain cytokines levels. We cannot exclude either a participation of peripheral immune organs and modification of blood immune cells and cytokines release to stroke response and to comorbid factors. Indeed, spleen and systemic immune response have shown contribution to the pathophysiology of stroke (Offner et al., 2006; Vendrame et al., 2006), diabetes (Tong et al., 2017), and hypertension (McMaster et al., 2015) that could impede neurorepair.

Untreated hypertensive mice presented a reduced astrocytes density in the peri-infarct area compared to non-hypertensive mice at D9 and, on the contrary, increased astrocytes density in treated hypertensive mice compared to untreated mice at D40. Moreover, untreated and treated hypertensive mice had increased astrocytes density between D9 and D40. Given the complex role of astrocytes following stroke, this increased glial scar at D40 is difficult to interpret as being a simple epiphenomenon or as a key event resulting in the absence of neurorepair (Morrison and Filosa, 2019).

Post-stroke dementia will eventually affect 25% of patients (Iadecola, 2013). This is a particular important heath issue since it is the second cause of cognitive decline after Alzheimer’s disease and that there is no current treatment to prevent or cure the disease. Still too few studies evaluate this aspect because of the need for models that must be maintained over time, complying with the increased early mortality when adding comorbidity (Poittevin et al., 2015). We found that diabetic mice with pMCAo presented altered spatial memory at Barnes maze but not non-diabetic pMCAO mice (Mangin et al., 2019). Structural alterations in the hippocampus, which is involved in information processing related to spatial memory, have already been observed after focal brain ischemia, with neurodegeneration in the CA1 zone in particular (Wang et al., 2004). Diabetes by itself can also decrease neuronal density (Artola et al., 2005; Amin et al., 2013) and impair long-term potentiation in rats (Artola et al., 2005). In our model, both conditions (pMCAo + diabetes) were necessary to alter the Barnes Maze, that also evaluates brain plasticity (Martin et al., 2000), but again hADMSC did not prevent spatial memory impairment in those mice. MSC beneficial effects rely partly on the promulgation of neurogenesis (Leu et al., 2010; Bao et al., 2011), limitation of neuronal death (Gutiérrez-Fernández et al., 2013), and angiogenesis promotion (Wang et al., 2008) none of which having been observed in treated mice. We failed to show spatial memory impairment in our hypertensive mice after 16 weeks of mild elevated blood pressure, although impaired vasoreactivity after 8 weeks was evidenced just as for diabetic mice. If the duration of the hypertension was appropriate to induce a sensorimotor deficit, we cannot exclude that both the duration of hypertension and/or the time points chosen to assess the Barnes maze test were inadequate and that a later evaluation would have been more contributive.

## Conclusion

These negative results are of utmost importance, given the importance of vascular risk factors in stroke patients. Comorbid animals should be part of all preclinical stroke studies and could avoid considerable unnecessary costs in clinical studies. The next preclinical study will focus on time administration and other models of vascular risk factors. If hADMSC prove then to be efficient, we will have the opportunity, by comparing the molecular pathways, to explore the in depths-mechanisms involved by these human ADMSC.

## Members of Resstore steering committee

O. Detante, Coordinator/Principal Investigator, Université Grenoble Alpes, Centre Hospitalier Universitaire Grenoble Alpes, Grenoble, France; E. Diez Tejedor (Principal Investigator, Servicio Madrileno de Salud, La Paz University Hospital, IdiPAZ Research Institute, Autonoma University of Madrid, Madrid, Spain; B. Fuentes, Servicio Madrileno de Salud, La Paz University Hospital, IdiPAZ Research Institute, Autonoma University of Madrid, Madrid, Spain, Madrid, Spain; M. Hommel, Université Grenoble Alpes Grenoble, France; A. Jaillard, Université Grenoble Alpes, Centre Hospitalier Universitaire Grenoble Alpes, Grenoble, France; J. Jolkkonen, University of Eastern Finland, Kuopio, Finland; R. Mikulik, St Anne’s University Hospital, Brno, Czechia; A. Moisan, Etablissement Français du sang, Saint-Ismier, France; F. Moniche, Neurology Department, Hospital Universitario Virgen del Rocio, Seville, Spain; J. Montaner, Vall d’Hebron Research Institute, Barcelona, Spain; A. Bustamante, Vall d’Hebron Research Institute, Barcelona, Spain; K. Muir, University of Glasgow, Glasgow, United Kingdom; H. Numminen, Pirkanmaa Hospital District, Finland; S. Miettinen, University of Tampere, Tampere, Finland; D. Koubi, Finovatis CEO, Lyon, France; C. Bollart, sponsor, Centre Hospitalier Universitaire Grenoble Alpes Grenoble, France. T. Keinonen, MEDFILES Oy CEO, Kuopio, Finland.

## Data Availability

The raw data supporting the conclusions of this manuscript will be made available by the authors, without undue reservation, to any qualified researcher.

## Ethics Statement

All experiments and surgical procedures were performed according to the European Community Directive (2010/63/EU), the ARRIVE (Animal Research Reporting In Vivo Experiments) guidelines, and the French national guidelines for the care and use of laboratory animals. The study was approved by the French ministry of Higher Education for Research and Innovation (APAFIS#5431-2016031912549126 v2).

## Author Contributions

GM performed the mice experiments including the acquisition and interpretation of data and drafted the manuscript. AC performed the analysis and interpretation of the data. AM provided the hADMSC. PB performed the Doppler experiments and analyses. BM performed experiments including the acquisition and interpretation of data. NK conceived and designed the study, and drafted and edited the manuscript. All authors read and approved the final manuscript.

## Conflict of Interest Statement

The authors declare that the research was conducted in the absence of any commercial or financial relationships that could be construed as a potential conflict of interest.
